# Liability for harm caused by AI in healthcare: an overview of the core legal concepts

**DOI:** 10.3389/fphar.2023.1297353

**Published:** 2023-12-14

**Authors:** Dane Bottomley, Donrich Thaldar

**Affiliations:** School of Law, University of KwaZulu-Natal, Durban, South Africa

**Keywords:** artificial intelligence, liability, Africa, healthcare, harm

## Abstract

The integration of artificial intelligence (AI) into healthcare in Africa presents transformative opportunities but also raises profound legal challenges, especially concerning liability. As AI becomes more autonomous, determining who or what is responsible when things go wrong becomes ambiguous. This article aims to review the legal concepts relevant to the issue of liability for harm caused by AI in healthcare. While some suggest attributing legal personhood to AI as a potential solution, the feasibility of this remains controversial. The principal–agent relationship, where the physician is held responsible for AI decisions, risks reducing the adoption of AI tools due to potential liabilities. Similarly, using product law to establish liability is problematic because of the dynamic learning nature of AI, which deviates from static products. This fluidity complicates traditional definitions of product defects and, by extension, where responsibility lies. Exploring alternatives, risk-based determinations of liability, which focus on potential hazards rather than on specific fault assignments, emerges as a potential pathway. However, these, too, present challenges in assigning accountability. Strict liability has been proposed as another avenue. It can simplify the compensation process for victims by focusing on the harm rather than on the fault. Yet, concerns arise over the economic impact on stakeholders, the potential for unjust reputational damage, and the feasibility of a global application. Instead of approaches based on liability, reconciliation holds much promise to facilitate regulatory sandboxes. In conclusion, while the integration of AI systems into healthcare holds vast potential, it necessitates a re-evaluation of our legal frameworks. The central challenge is how to adapt traditional concepts of liability to the novel and unpredictable nature of AI—or to move away from liability towards reconciliation. Future discussions and research must navigate these complex waters and seek solutions that ensure both progress and protection.

## 1 Introduction

Modern artificial intelligence (AI) is the cornerstone of the fourth industrial revolution. Successes in data availability, algorithm design, and processing power (Craglia et al., 2018) have empowered AI systems to make dramatic impacts in disparate sectors including transportation, education, agriculture, public services, finance, and healthcare (Artificial Intelligence for Africa: An Opportunity for Growth, Development, and Democratisation, 2018).

The varying degrees of autonomy with which AI systems can operate distinguish it from other emerging technologies. The advantage of AI lies in its ability to process massive amounts of varied information, and thereby perform valuable functions or draw useful conclusions inspired by its interpretation of the information. However, the essence of its usefulness is also its most challenging feature. For example, machine learning is a common approach to AI system design in medicine. Instead of programming the system for all possible scenarios with specific instructions, when using machine learning, developers set a broad goal which the system uses to form its own instructions to achieve the goal through repeated experiments and self-research (Rachum-Twaig, 2020). As it processes information, the AI system adjusts the parameters by which it judges inputs to produce more accurate outputs, effectively programming itself (Townsend, 2020). These approaches usually produce more accurate systems and they also require less human control (Grimm et al., 2021). Alarmingly, this and similar approaches to AI system design remove the human element at key stages of development in a way which may complicate inquiries into the attribution of responsibility and liability. This becomes especially pronounced where the AI system is so complex that its operations become inscrutable to humans. These so-called “black-box” algorithms lack the transparency to fully audit how they came to the conclusions they did. In response to this issue, some developers have endeavoured to design and create ‘explainable’ AI systems and ways of ensuring transparency which would foster an environment of accountability and responsibility and create better evidence when determining liability (Ali et al., 2023).

Determining responsibility will be important in dealing with the social challenges of AI integration. Perc et al. (2019) investigates how AI systems will likely have to choose between acting in favour of one party’s interest over another in certain contexts and how this may influence how the technology evolves. Developers may be incentivised to produce systems which favour owners’ interests above users’ in order to drive sales. The solution may be to require that AI systems act in the interests of the broader community; however, this policy may create its own issues in that it will potentially disincentivise people from buying AI systems which will not protect their interests outright and therefore lead to a lower adoption and investment in AI systems overall. This approach may then fail to fully realise the safety gains which can be had by increased AI usage. Of course, as Perc et al. (2019) consider, another approach may be to leave such decisions for the AI system to decide itself, or simply leave it to chance. This approach, however, suffers from a lack of clear answers to questions of responsibility and liability for the outcomes of decisions. Robust regulation and thoughtful juristic approaches to AI challenges will be necessary to provide adequate responses to responsibility for actions in these cases. This will be vital to supporting the benefits of AI integration whilst properly addressing the risks of the technology. Specifically, in healthcare, AI systems show impressive potential to increase the overall efficiency of healthcare systems and to manage disease outbreaks (Owoyemi et al., 2020). Furthermore, these systems can increase the reach of initiatives, while supplementing an already overburdened sector (Pepper and Slabbert, 2011). However, healthcare institutions deal with patients who are at their most vulnerable, where an incorrect decision could prove fatal. In addition, healthcare practitioners are required to abide by particularly high ethical and legal standards which AI systems may not easily conform to. In particular, the black box nature of some algorithms may prevent physicians from providing enough information to their patients about their treatments to satisfy requirements of informed consent, the emergent abilities of AI systems also raise questions as to how they will be considered in relation to the usual standard of care expected of physicians, and medical liability may need to be redefined for AI use.

Many jurisdictions already have laws and regulations which would encompass AI technologies; however, the specific challenges of AI may mean that these regulations do not provide desirable results when they are relied upon. As a response to this, many jurisdictions outside Africa have begun drafting specific AI law and regulations (Sallstrom et al., 2019). Providing a proper response to the issues posed by AI use in healthcare is essential to providing legal certainty to all stakeholders. This will allow them to order their interactions with AI systems and create an environment of trust in relation to AI use (European Commission, Directorate-General of Communications Networks, Content and Technology, 2019). This trust will be important for the future of AI as a lack of trust could permanently harm the reputation of AI in healthcare, or lead to additional costs through inefficient regulation or repeated amendment (Floridi et al., 2018).

The aim of this article is to set the stage for legal development and policy initiatives in Africa by exploring the legal concepts relevant to the attribution of liability for AI harm. First, we begin by describing current developments and the use of AI in healthcare in Africa in Section 2. Then we discuss the concept of liability broadly in Section 3. In Section 4, we describe how AI presents novel challenges to liability determination, particularly the concept of personal liability. In Section 5, we review the different approaches to determining liability. We provide our concluding thoughts in Section 6.

## 2 Artificial intelligence in healthcare in Africa

AI systems in healthcare can perform tasks normally requiring human physicians (Joshi. and Morley, 2019). Most current uses are in diagnosis and screening; however, future systems could scan images, discover new drugs, optimise care pathways, predict positive treatment outcomes, and provide preventative advice (Joshi and Morley, 2019). Increased use of AI allows physicians to focus on tasks where, given the current state of technology, they cannot be replaced. Furthermore, AI could further broaden public health initiatives by increasing access and tracking disease outbreaks, while lowering the cost of care (Joshi and Morley, 2019).

For example, DeepMind’s AlphaFold is an AI system which accurately predicted the protein structures of the COVID-19 virus, being an important aspect of creating a vaccine (Jumper et al., 2020). This use could greatly reduce vaccine response times in the future. IBM’s Watson for oncology is another system which has been able to analyse genomic data of patients in light of medical data from vastly more journals than a person could process, so providing more personalised treatments with high accuracy rates (Chung and Zink, 2018).

While other jurisdictions are considering policy-level AI implementation in healthcare systems (Joshi,and Morley, 2019), Africa has had relatively little meaningful interaction with AI in healthcare both academically (Tran et al., 2019) and clinically (Owoyemi et al., 2020) and, currently, African countries are at a nascent stage in their AI regulatory policies (Townsend et al., 2023). This is despite AI’s utility in developing countries where AI systems could lead to better utilisation of resources and enable new, effective treatments and treatment management systems (Sallstrom et al., 2019). Furthermore, AI systems can provide overarching and effective treatment options that improve standards of living, improve direct patient care, maximise supply-chain efficiencies, reduce administrative tasks, and streamline and improve compliance measures (Sallstrom et al., 2019).

Even though relatively limited, there has been some AI system use in Africa. In South Africa, Vantage, a machine learning-based system developed by BroadReach Healthcare, was used to assess clinics’ performance and provide staffing and operational recommendations in HIV clinics in KwaZulu-Natal (Singh, 2020). Further, DrConnect, an application by Discovery Health, provides personal assessments of medical symptoms and advice and remote support using AI technology, by using information from wearable devices such as smartwatches, to give medical and lifestyle advice (Singh, 2020). In Ghana, MinoHealth AI labs have used AI systems for automated diagnostics, forecasts, and prognostics. Also, BareApp is using specialised AI technology to diagnose skin disease and suggest treatments (Eke et al., 2023). In Uganda, AI is being merged with other technologies to develop a specialised system in the management of female chronic diseases (Eke et al., 2023). In Nigeria, Ubenwa is using AI to improve the diagnosis of birth asphyxia in low-resource settings (Owoyemi et al., 2020). Also in Nigeria, AI is proving effective in the identification of fake drugs (Owoyemi et al., 2020).

These examples illustrate the growing use and development of AI systems in Africa. However, as this use grows, it will be vital that African countries position themselves to take full advantage of AI’s benefits. Legal regulation will be especially important in directing AI system use and development by providing legal certainty by the formation of proper policies and regulations. A main concern though will be the determination of liability for AI harm.

## 3 Understanding liability

The nature of emerging technologies is that we need time to understand them and develop policies and regulations which will encourage equitable use (Calo, 2015). AI in healthcare is no different. While AI has the potential to positively influence healthcare, its implementation must necessarily be coupled with appropriate safeguards to minimise risks of harm (European Commission, Directorate-General for Justice and Consumers, 2019). Specific to AI, unforeseeable risks may still arise in apparently well-trained systems where performance is being improved (World Health Organisation, 2021). As it currently stands, when risks arise, our existing policies and regulations will be the basis of determining who is responsible and liable for the harm caused. Assessing whether these policies and regulations are sufficient to properly determine responsibility will be important, as the determination of responsibility plays an important role in determining the basis of legal liability for AI conduct and garnering trust in AI usage more broadly. Currently, this will largely depend on civil liability rules.

Generally, civil liability provides the dual purpose of providing a means for victims of harm to be compensated, while also providing an economic incentive for those held liable to avoid continuing harmful conduct (Buiten et al., 2021). Accordingly, these rules are an important means of protecting patients and providing clarity to businesses on how they may innovate and operate their products (Buiten et al., 2021). However, the varying complexity of AI systems, system updates, algorithms which change from environmental input, and cyber-security concerns may make it difficult to justify claims for compensation and to provide clear pathways for victims to bring claims (European Commission, Directorate-General for Justice and Consumers, 2019). It is also unclear whether the rationale behind current liability regimes will be effective in dealing with AI harm. For example, where AI systems make decisions, it may be difficult for a plaintiff to find a suitable defendant or for a court to determine the standard of care to be expected from an AI system. Therefore, it is currently unclear how current liability regimes will consider AI harm in healthcare.

Proper liability policy formation will consider the outcomes of current liability rules but, in addition, it must necessarily consider the impact which the policy will have on the development and use of AI in the future. This means tailoring policy towards managing AI-specific risks while encouraging positive uses. For example, a lack of legal certainty and fear of unreasonable legal penalties for relying on AI recommendations may discourage healthcare practitioners from using AI systems as active participants in treatment, relegating AI systems’ role to the mere confirmation of decisions made by healthcare practitioners (World Health Organisation, 2021). On the contrary, removing penalties may encourage AI systems use; however, this position may be tenable only where existing issues of accountability and responsibility are properly considered.

Of particular concern in healthcare should be determining how an AI system will form part of the standard of care. Such a determination will be essential for providing sufficient information for physicians and patients to make decisions about relying on the technology (World Health Organisation, 2021). The decision of the physician is important as he/she will also likely be responsible for the proper operation, monitoring and maintenance of the technology (Bertolini and Episcopo, 2021), and their decision could be consequential for their employer through vicarious liability (World Health Organisation, 2021).

A concern specific to Africa is that many policy frameworks which would guide the development of AI systems are created in environments outside of Africa. Moreover, a lack of access to high quality data sets and limitations in infrastructure could lead to the use of algorithms which are predominantly developed outside of Africa. These could be potentially prejudicial as they may not be properly designed to work in low-resource environments (World Health Organisation, 2021). Therefore, liability policies will need to consider that developers are situated outside of Africa, and that algorithms are adapted for, rather than designed for, the African context.

The role of an AI policy framework should be to prevent AI harm and to promote AI innovation, following a risk-based, rights-preserving, agile, adaptive, and innovation-supporting regulatory approach (Townsend et al., 2023). Robust and effective regulation will provide important guiding principles for the development and implementation of AI systems in healthcare in Africa (World Health Organisation, 2021). Legal certainty will provide routes for compensation for patients and ensure accountability and responsibility through integration and innovation in the healthcare system.

## 4 Challenging our understanding of liability: AI and personhood

AI systems’ successful imitation of qualities normally associated with humans has bolstered the inquiry into AI personhood (Abbott and Sarch, 2019). A crucial development in support of AI personhood has been the ability to program generalised goals into AI systems. This approach is markedly different to traditional software as the AI system is programmed to decide what steps it would take to achieve its goal, instead of being programmed with specific, step-by-step instructions (Bostrom and Yudkowsky, 2014). This goal-directed behaviour is what powered IBM’s Deep Blue chess robot. Programmers surpassed their own chess skills by encoding the rules of the game into Deep Blue and relying on its superior processing power to find ways of “winning” which the developer would not be able to do (Bostrom and Yudkowsky, 2014). Should this be enough to draw the necessary philosophical conclusions on AI personhood, it is clear that the legal implications would be substantial (Solum, 1992). Where AI systems are considered persons, even in limited form, they may be held responsible for their actions in their own capacity.

However, the utility of recognising AI personhood should not replace thoughtful policy formation. An AI system fulfilling roles normally delegated to humans does not mean that personhood necessarily follows (Thaldar and Naidoo, 2021). This may be illustrated by the recent granting of a patent in South Africa where the sole inventor was AI. Although some would consider “inventing” to be a human characteristic, without the ability to fully contain human emotion and capacity to engage in relationships, it is difficult to see such an AI system as more than a “special species of legal object that has the ability to invent” (Thaldar and Naidoo, 2021). As AI becomes more autonomous, legal rules can be developed to allow for special treatment of AI systems, which would be comparable to the legal rules that provide for the special treatment of animals (Thaldar and Naidoo, 2021).

While it is generally agreed that current AI systems are not capable of being considered legal persons, more sophisticated, generalised, and autonomous systems may change this assumption (Solum, 1992). Current systems can be changed, created, or completely deleted like any other software, but where AI systems enjoy a degree of personhood, our relationship with them may become far more complicated. Legally, the granting of AI personhood would aid plaintiffs of AI harm in that they could gather evidence from the AI system through its examination as a witness (Chung and Zink, 2018). However, this benefit may be somewhat limited in systems that lack transparent reasoning.

More definitively, some scholars insist that a separate legal personality for AI systems will never be necessary (European Commission, Directorate-General for Justice and Consumers, 2019). They contend that even fully autonomous systems’ actions are better attributed to individuals or other legal persons than to the system itself (European Commission, Directorate-General of Communications Networks, Content and Technology, 2019).

An important consideration is that AI systems’ lack of abstract thought limits their comparison to human personhood and decision-making, particularly in healthcare. Whereas human decision-making in healthcare is largely justified by morality, AI systems lack moral input in decision-making (Chung and Zink, 2018). Moral considerations become vitally important in healthcare and resource-scarce environments where circumstances require difficult decisions to be justified, usually with reference to moral ideals. Therefore, we suggest that in lacking moral capacity, AI systems could be limited in how they could be held accountable if they were considered persons or could lack prerequisites to make decisions in moral contexts.

For scholars who consider AI more than a tool, the lack of moral input is an issue they contend with (Bashayreh et al., 2021). Dignum (2017) suggests that even AI systems acting as assistants may inherit a moral framework for decision-making through incorporating the values of their engineers. However, a mere copy of an engineer’s morals may not necessarily lead to satisfactory results as AI systems may not apply moral lessons to their environments in the same way as humans (Bostrom and Yudkowsky, 2014). Dignum contends that identifying and analysing these imbued values will nevertheless improve system performance (Dignum, 2017). This would also ensure that incorporated morals are interpreted in an acceptable way, meaning that, as these systems become more autonomous and powerful, moral assessment may become an essential component of their decision-making, especially in a field such as healthcare (Dignum, 2017).

Accordingly, there is some possibility of future AI systems bearing some form of personhood (Solum, 1992). However, conferring even a limited form of personhood on AI systems presents further practical difficulties. For example, as is commonly suggested, a limited form of personhood may be imbued on AI systems through the extension of the principal-agent relationship. In determining responsibility, however, the standards which apply when adjudicating AI system conduct and under what circumstances AI systems would be considered liable for their conduct would remain unclear. This will be discussed further in the section on the principal-agent relationship below.

Further, a final practical issue of attributing liability directly to AI systems is that it leaves no clear pathways for compensation of victims (Bashayreh et al., 2021). As AI systems are currently incapable of ownership, there are no assets that a victim could claim. To remedy this situation, some scholars have suggested the introduction of an insurance scheme funded by developers from which victims may claim (European Commission, Directorate-General of Communications Networks, Content and Technology, 2019). However, such a scheme may not adequately replace clear and fair liability rules and could lead to high administrative costs, so defeating the cost-saving benefits of a clear claim process (European Commission, Directorate-General of Communications Networks, Content and Technology, 2019). Furthermore, there is a lack of guidance on the value of AI insurance policies as there are no standards against which to assess risk or begin a cost analysis (Bertolini and Episcopo, 2021).

## 5 Approaches to attributing liability

The subsections below discuss the main approaches to the attribution of liability for harm caused by AI systems in healthcare. Section 5.1 broadly considers the extension of the principal–agent relationship to include AI systems and the consequences of such an extension. Section 5.2 deals with AI as a product and how consumer protection law standards may be applied to AI system harm. We then comment on current fault-based liability regimes as they apply to AI systems in Section 5.3. This leads to a discussion of efforts to use strict liability to attribute liability for AI harm in Section 5.4. In Section 5.5, we consider an approach to AI harm focusing on improving AI system use in healthcare through reconciliatory forums.

### 5.1 Principal–agent relationship

Most current AI systems in healthcare act as assistants to healthcare practitioners (Joshi and Morley, 2019). Accordingly, some scholars have suggested extending principal–agent rules to govern liability (Rachum-Twaig, 2020). This approach is mostly modelled after the doctor–medical student relationship whereby a medical student performs tasks under the authority and supervision of a doctor; however, the doctor attracts liability for harm which occurs during the student’s duties (Chung and Zink, 2018). IBM’s Watson operated under a similar regime, whereby the system would assist physicians in making decisions and providing recommendations; however, the physician carried responsibility for the final decision (Chung and Zink, 2018). This approach would ensure that there is always an identifiable human part of the decision-making process and would be in line with an AI design philosophy called “human-in-the-loop” systems (HITL) (Dignum, 2017). HITL ensures proper oversight of system decisions, while creating a clear party to hold accountable by making a human ultimately responsible for decisions (Dignum, 2017).

Although this approach provides a justification for attributing liability to a specific person, it may disincentivise practitioners from following system recommendations as they would bear the risk of harm. The tension arises where the physician may not be able to understand how the system came to its decisions and therefore be unable to assess the risk of harm himself or herself. He or she will likely, however, justify considering AI recommendations based on AI’s profound ability to consider vastly more information than he or she could. This could potentially lead to increased costs of medical care and slower treatments as practitioners seek alternative means of validating their decision to follow or reject AI system recommendations. This may be so until there is guidance as to AI systems’ position in the standard of care. Should AI systems form part of the standard of care, there may be an expectation for physicians to follow AI recommendations, potentially only until they have a clear professional duty to act otherwise.

Furthermore, similar to criticism of AI personhood, critics of HITL argue that there is a difficulty in determining the correct standard against which to compare the conduct of the AI system (Kingston, 2016). Initial systems may be comparable to humans; however, as systems begin to outperform humans, another standard may need to be considered (European Commission, Directorate-General of Communications Networks, Content and Technology, 2019). In addition, as systems become more sophisticated, there remains uncertainty as to how disagreements between AI system recommendations and human practitioner recommendations should be resolved. Current norms suggest that claims for damages will favour standard care pathways, even where AI systems recommend non-standard treatments (Tobia et al., 2021). This seems to be true, regardless of the outcome of treatment and healthcare practitioners are more likely to attract liability where they do not follow these standards (Price et al., 2019). This initial bias against non-standard care could limit growth of AI technology use in healthcare, which could limit future AI development as there will be a lack of testing in a medical environment and a lack of opportunity to build trust (World Health Organisation, 2021).

Importantly, healthcare practitioners could be less willing to implement recommendations for AI systems which deviate from standard care procedures where they face liability for acting on AI recommendations. However, as AI systems become commoner in healthcare, the bias against their inclusion could shift, especially where AI systems become part of the standard of care (World Health Organisation, 2021). The attribution of liability to the developer of the system may follow if they are in the best position to prevent harmful outcomes as the creator of the system (Lövtrup, 2020).

### 5.2 Product liability

Townsend et al. (2023) found that eleven out of twelve African countries surveyed provide for strict liability of harmful or defective goods in their consumer protection laws. Therefore, anyone in the supply chain could in principle be held strictly liable for AI harm to the patient. However, are these consumer protection laws sufficiently equipped to deal with AI-specific risks? Core to consumer protection law is the concept of a product *defect*. To attenuate strict liability, it must be proven that a product had a defect. However, the inherent unpredictability of AI systems makes it difficult to define what constitutes a defect in the context of AI (Bashayreh et al., 2021). The South African Supreme Court of Appeal held that a consumer who is claiming in terms of South Africa’s Consumer Protection Act (South African Government, 2009) must prove not only the existence of a defect, but also that the defect is material (Motus Corporation, 2021). Furthermore, it is difficult to prove that a defect caused harm (European Commission, Directorate-General of Communications Networks, Content and Technology, 2019), or that the developer was responsible for the defect (European Commission, Directorate-General of Communications Networks, Content and Technology, 2019). When using multiple systems together, as is common in healthcare, attributing fault may be impossible (European Commission, Directorate-General of Communications Networks, Content and Technology, 2019). Modern regulations were drafted before the AI boom, and therefore are unlikely to have properly considered AI-specific issues (Lövtrup, 2020). Accordingly, patients who have suffered harm caused by AI are likely to face a considerable evidentiary burden when seeking resolution through product liability law.

In the United States, software has generally been considered a tool and courts have been hesitant to extend product liability to healthcare software developers (Gerke et al., 2020). In Europe, the “developmental risk defence” allows a producer to avoid liability on the basis that scientific knowledge at the time of production was unable to detect the existence of a defect in the product (Holm et al., 2021). Sihlahla et al. (2023) note that in South Africa, a healthcare practitioner or a healthcare establishment sued in terms of the Consumer Protection Act (South African Government, 2009) for harm caused by AI would have a complete defence if they can show that they could not reasonably have been expected to have discovered the defect.

### 5.3 Fault-based remedies

Generally, fault-based liability is based on a person’s intentional or negligent conduct which causes harm wrongfully and culpably (Mukheibir et al., 2010). Liability is attributed based on a determination of who should justly compensate for the damages of the plaintiff (Marchisio, 2021). Currently, there is no case law to guide the application of fault-based liability principles, particularly in cases where the AI suffers from an unknown flaw which was not reasonably foreseeable (Donnelly, 2022).

Accordingly, key elements of such remedies are difficult to prove in AI system cases, specifically causation and fault. Causation is difficult to prove as it may be difficult to show a flawed algorithm was the cause of harm (European Commission, Directorate-General of Communications Networks, Content and Technology, 2019). Similar to product law, it may be difficult to determine what a flaw is, or at what point the flaw was created if the system was developed by multiple parties (European Commission, Directorate-General of Communications Networks, Content and Technology, 2019). Even where a flaw is identified, demonstrating foreseeability for negligence-based claims is still difficult (Holm et al., 2021). Furthermore, establishing vicarious liability would be complicated as, currently, there is no means of determining whether the AI system “acted negligently” or what degree of control a medical practitioner should exert over an AI system (Donnelly, 2022). Accordingly, where there is no causation on the part of the physician, a patient may be left with no recourse (Donnelly, 2022).

Fault-based liability is an important means of deterrence (Buiten et al., 2021). Defendants who are penalised are incentivised to prevent harm in the future (Marchisio, 2021). This is justified as the defendant should be the one best oriented to assess and avoid risk (Marchisio, 2021). However, AI systems’ necessary unpredictability may make it impossible for a particular party to act to prevent harm as it would be unforeseeable.

Therefore, it has been suggested that liability, by rule, be shared among the technical and medical stakeholders as part of their joint contribution to the risk of harm in the use of the system (Smith and Fotheringham, 2020). This could be in the form of joint and several liability or proportional liability (European Commission, Directorate-General of Communications Networks, Content and Technology, 2019) using the person’s choice to develop or implement the system as the justification to establish causation (Bashayreh et al., 2021).

An extension of this idea is a risk-sharing approach (Bashayreh et al., 2021). Owners and developers would bear liability proportionate to the risk each has accepted in their role in the AI lifecycle, operating to the exclusion of cases of wilful misconduct or gross negligence (Bashayreh et al., 2021). Importantly, developers would need to disclose all risks and potential deficiencies of the system, including the degree to which the system’s decision can be explained and all the built-in values of the system (Bashayreh et al., 2021). In addition, owners would disclose their intended use of the product and the environment it will be deployed in (Bashayreh et al., 2021). In the event of harm, liability could be portioned by a court adjudicating on the facts with relevant disclosures.

The creation of responsibilities at different stages of the AI system’s lifecycle remains a common approach to justifying liability in fault-based approaches in literature. Current fault-based standards already attach responsibilities to people based on special relationships they may have with an object, such as where the person is in control of a potentially dangerous animal or thing (Marchisio, 2021). Where the animal acts unpredictably, the person controlling it could be held liable (Bashayreh et al., 2021). Failure to fulfil responsibilities to protect others from harm in this type of relationship will justify the attribution of liability. This approach may be useful in AI through the prescription of minimum rules to establish wrongfulness and fault (European Commission, Directorate-General of Communications Networks, Content and Technology, 2019). Where these standards are not upheld, the burden of proof may shift in favour of the victim. Therefore, Rachum-Twaig (2020) suggests the creation of “safe harbours.” Safe harbours act as points in the AI lifecycle where a party is responsible for ensuring certain minimum standards. Where the party fails to uphold these standards, they are more likely to incur liability and current fault-based remedies can be employed. Approaches like this form part of a movement towards risk-based liability replacing the foreseeability element of many fault-based regimes (Calo, 2015).

### 5.4 Strict liability

The clear issues that arise in justifying attribution of liability to certain stakeholders has encouraged some scholars to suggest no-fault or “strict” liability systems as better means of attributing liability (Holm et al., 2021). No-fault liability makes it significantly easier for victims to claim compensation by providing clear pathways to settle claims and removing the necessity of proving fault (European Commission, Directorate-General of Communications Networks, Content and Technology, 2019). This eases the burden on claimants who are already the victim of harm when reporting errors and provides better hope of reconciliation (Holm et al., 2021). No-fault systems also separate the compensation and liability claims (Holm et al., 2021). They remove the necessity of victims to access information to prove fault, which is a particular concern with inscrutable AI systems. The occurrence of harm is made the centre of the claim instead of proving fault.

Concerns raised about this approach have focused on the future development of AI systems (European Commission, Directorate-General of Communications Networks, Content and Technology, 2019). First, strict liability would subject stakeholders to material burdens with no fair opportunity to avoid them (Abbott and Sarch, 2019). Normally, strict liability applies for unexpected harms, but where AI systems are implemented, it is difficult to determine how unexpected harms would be defined, as the systems are necessarily programmed to be unpredictable (European Commission, Directorate-General for Justice and Consumers, 2019). Second, stakeholders would be at risk of reputational damage resulting from the occurrence of harm which is otherwise not foreseeable (Abbott and Sarch, 2019). Therefore, stakeholders would be subject to significant burdens without an opportunity to take effective measures against the realisation of these harms.

To ease the potential economic impact stakeholders may experience under strict liability, it has been suggested that a stakeholder-funded scheme be created to compensate victims of AI harm (European Commission, Directorate-General for Justice and Consumers, 2019). This may further simplify the pathways for victims to claim; however, a mixed fund would lead to innocent parties effectively being held liable for harm they did not cause (European Commission, Directorate-General for Justice and Consumers, 2019). Furthermore, the burden on blameworthy parties would be eased as they would pay only a portion of any damages claims for harm caused by their systems. This reduction would add to the already perceived loss of the deterrent effect as litigation is no longer available to claimants (European Commission, Directorate-General for Justice and Consumers, 2019). One suggested solution is to model the New Zealand approach to medical matters whereby no-fault systems have been implemented in certain medical matters, but claims are limited to unusual injuries (European Commission, Directorate-General for Justice and Consumers, 2019).

Practically, strict liability could potentially be more expensive than litigation when administrative costs are coupled with more patients being eligible to claim (Holm et al., 2021). Also, a strict liability system may not be capable of being applied cross-jurisdictionally or globally (Rachum-Twaig, 2020). This has led some scholars to suggest that a mixture of fault and no-fault rules could provide equitable AI regulation (Marchisio, 2021).

### 5.5 Reconciliation

The adversarial nature of the approaches to liability outlined above may be counter-productive to the proper regulation of AI technology—at least during its nascent stage. Naidoo et al. (2022) argue that instead of prioritising questions such as “Who acted?” and “Was the act wrongful?,” which causes persons involved to be antagonistic and defensive, the focus should shift to (a) learning how to better use AI in healthcare, and to (b) actively developing guidelines for AI developers and healthcare professionals who are using AI systems. The authors suggest that (a) and (b) can best be attained by establishing a *sui generis* dispute resolution institution for harm caused by AI in healthcare. This institution would replace litigation in the courts, hold broad investigative powers to access all relevant information, resolve disputes through reconciliation, award financial redress to victims of AI-driven harm in healthcare, and—importantly—learn and develop guidelines. In essence the authors argue for reconciliation to replace litigation as they view reconciliation as more conducive to the learning element of a regulatory sandbox.

This approach could draw inspiration from current alternative dispute resolution structures, principally, the South African Commission for Conciliation for Conciliation, Mediation and Arbitration (CCMA). The compensation structure could draw lessons and inspiration from the operation of the South African Road Accident Fund which compensates victims of accidents on public roads for bodily harms. The basis of this system could encompass a more inquisitive approach to litigation, whereby all parties are enabled to share information with the institution taking a more active role in discovery through its investigative powers. A thoughtful use of the institution’s powers to adjudicate the matter can help to ensure that power disparities between the parties could be mitigated whilst providing for a just outcome.

The guidelines developed by the *sui generis* dispute resolution institution can over time either become customary law in the field, or be solidified in legislation—depending on the preferences and traditions of the relevant jurisdiction. This would signal that AI technology and the regulation thereof has reached a stage of maturity, at which stage the *sui generis* dispute resolution institution would have served its purpose, and a return to a liability-based approach can be considered.

## 6 Conclusion

The assimilation of AI technologies in the African healthcare sector is an unprecedented juncture in the continent’s journey towards equitable and advanced medical care. As AI solutions make inroads into African medical establishments, they bring along a multitude of autonomy and opacity issues, challenging the longstanding ethical pillars and legal norms ingrained in the diverse cultures of the continent. The quintessential medico-legal principle of informed consent is now juxtaposed against the intricate algorithms of AI, challenging the very essence of transparency and patient understanding. Similarly, the increasing autonomy of AI systems amplifies the intricacies of liability, pushing the boundaries of traditional legal frameworks.

In this article, we tried to provide the reader with an overview of the legal concepts relevant to the issue of AI and liability in healthcare. We started with the contemplation of AI personhood. While captivating, we suggest that it poses substantial challenges in an African context, particularly when addressing tangible redress mechanisms for AI-induced mishaps. Next, the principal-agent framework, although providing a modicum of accountability, could inadvertently stifle the AI adoption rate by placing considerable responsibilities upon local medical practitioners. While product liability law offers another plausible approach, it struggles to categorise the continually evolving nature of AI in the static confines of conventional product definitions. Alternative strategies, such as risk-based liability may offer clearer paths in contexts where fault determination proves onerous. Yet, they too grapple with ensuring specificity and justice. Strict liability, offering more transparent compensation mechanisms, raises concerns about economic implications, reputational risks and, most critically, the challenge of harmonising such policies across Africa’s diverse legal landscapes.

An approach based on reconciliation rather than liability potentially provides the best environment for a regulatory sandbox; however, reconciliation in the context of AI-driven harm in the healthcare context lacks the same level of scholarship as the approaches based on liability. We suggest that reconciliation offers much potential and deserves more academic attention.

In distilling these insights, it is evident that Africa’s AI journey in healthcare is not solely a scientific or medical transition. It also requires profound legal reflection and evolution.
