# Corrigendum: Liability for harm caused by AI in healthcare: an overview of the core legal concepts

**DOI:** 10.3389/fphar.2024.1366004

**Published:** 2024-03-14

**Authors:** Dane Bottomley, Donrich Thaldar

**Affiliations:** School of Law, University of KwaZulu-Natal, Durban, South Africa

**Keywords:** artificial intelligence, liability, Africa, healthcare, harm

In the published article the **Acknowledgment** section was omitted. The **Acknowledgment** should read:

## Acknowledgment

The authors acknowledge the use of ChatGPT4 from Open AI to improve the language and readability of the abstract, introduction and conclusion sections of this article.

The authors apologize for this error and state that this does not change the scientific conclusions of the article in any way. The original article has been updated.

